# Successful desensitization with human insulin in a patient with an insulin allergy and hypersensitivity to protamine: a case report

**DOI:** 10.1186/1752-1947-2-283

**Published:** 2008-08-26

**Authors:** Claudia Pföhler, Cornelia SL Müller, Dirk O Hasselmann, Wolfgang Tilgen

**Affiliations:** 1 The Saarland University Hospital, Department of Dermatology, 66421 Homburg/Saar, Germany

## Abstract

**Introduction:**

Insulin allergy may occur in patients treated with subcutaneous applications of insulin preparations. Besides additives in the insulin preparation such as protamine, cresol, and phenol, the insulin molecule itself may be the cause of the allergy. In the latter case, therapeutic options are rare.

**Case presentation:**

A 68-year-old man with poorly controlled type 2 diabetes mellitus received different insulin preparations subcutaneously while on oral medication. Six to eight hours after each subcutaneous application, he developed pruritic plaques with a diameter of >15 cm at the injection sites that persisted for several days. Allergologic testing revealed positive reactions against every insulin preparation and against protamine. Investigation of serum samples demonstrated IgG antibodies against human and porcine insulin. We treated the patient with human insulin using an ultra-rush protocol beginning with 0.004 IU and a rapid augmentation in dose up to 5 IU. Therapy was accompanied by antihistamine therapy. Subsequent conversion to therapy with glargine insulin (6 IE twice daily) was well-tolerated.

**Conclusion:**

As reported in this case, desensitization with subcutaneously administered human insulin using an ultra-rush protocol in patients with an insulin allergy may present an easy form of therapy that is successful within a few days.

## Introduction

In the past, when unpurified insulins were used, allergic reactions to the drug were reported in 10% to 56% of patients [1]. Since human insulin and its analogues have been introduced, insulin allergies are rare and currently reported in only 0.1% to 2% of all patients treated with insulin [2]. In most cases, allergic reactions are restricted to the skin and are either of a local immediate or delayed reaction type. These skin reactions are often self-limited under continuation of therapy. However, systemic, potentially life-threatening reactions such as urticaria or anaphylaxis have also been reported [1]. Both types of hypersensitivity may result from the insulin molecule itself, and also from protamine, which is used in many preparations to delay insulin absorption [3-5]. Protamine sulphate is a low-molecular weight polycationic protein isolated from sperm of salmon or salmon-like fish. Besides its use as an insulin additive, protamine is also used to reverse the therapeutic effects of heparin. The intravenous or subcutaneous administration of protamine can provoke pseudoallergic reactions through non-immune mediated histamine release [5]. In patients with diabetes mellitus, subcutaneous administration of protamine-containing insulin preparations can also provoke delayed, T-cell mediated skin reactions or granulomatous hypersensitivity [6]. In addition to protamine, cresol and phenol, which both serve as preservatives in pharmaceutical products, may provoke allergic reactions [7].

Successful treatment of insulin allergies has been reported using a continuous subcutaneous pump infusion of insulin [8-10], switching from human insulin to insulin aspart or lispro [11,12], or in severe cases, by pancreas transplantation [13,14].

In the case presented, we suggest tolerance induction using an ultra-rush desensitization protocol as an easy-to-perform and well-tolerated therapy for patients with insulin allergies.

## Case presentation

We evaluated a 68-year-old man in our dermatologic outpatient unit. He suffered from type 2 diabetes and was initially treated with oral anti-diabetic medication. As normoglycaemia was not being achieved using maximal oral treatment and a low caloric diet, the patient was treated with insulin. The administration of different insulins (i.e. insulin detemir, insulin glargine, and human insulin) resulted in the development of pruritic plaques with a diameter of >15 cm at each injection site and which persisted for several days. Splitting of the dose and changing of the injection sites were not successful in resolving the reaction. Local factors, such as poor injection technique, misuse of the insulin injector, incorrect use of local disinfectants, or contact allergy to disinfectants were ruled out.

### Skin tests

**Figure 1:**
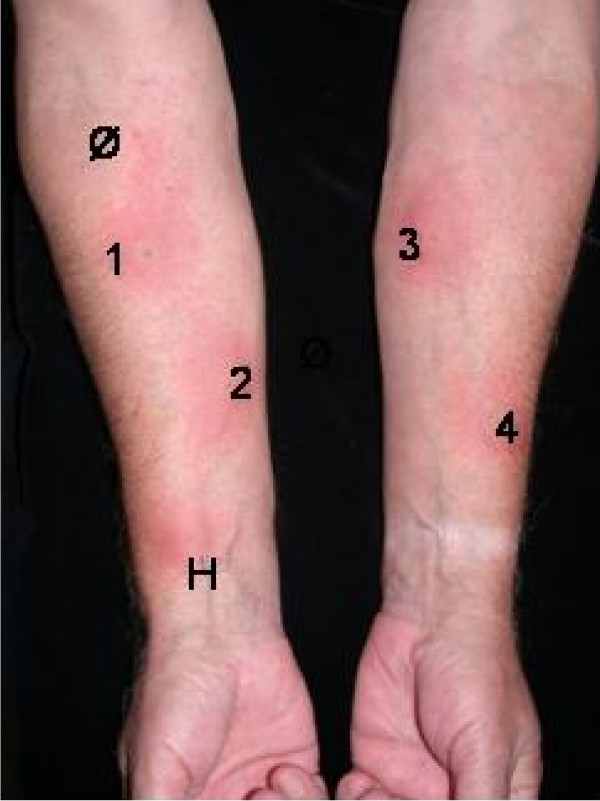
**Intradermal testing showing positive reactions against *Levemir^© ^(1), Huminsulin basal^© ^(2), Humalog^© ^(3)*, and *Lantus^© ^*(4) 20 minutes after injection**. Histamine (H) served as a positive, aqua dest. (Ø) as a negative control.

**Figure 2:**
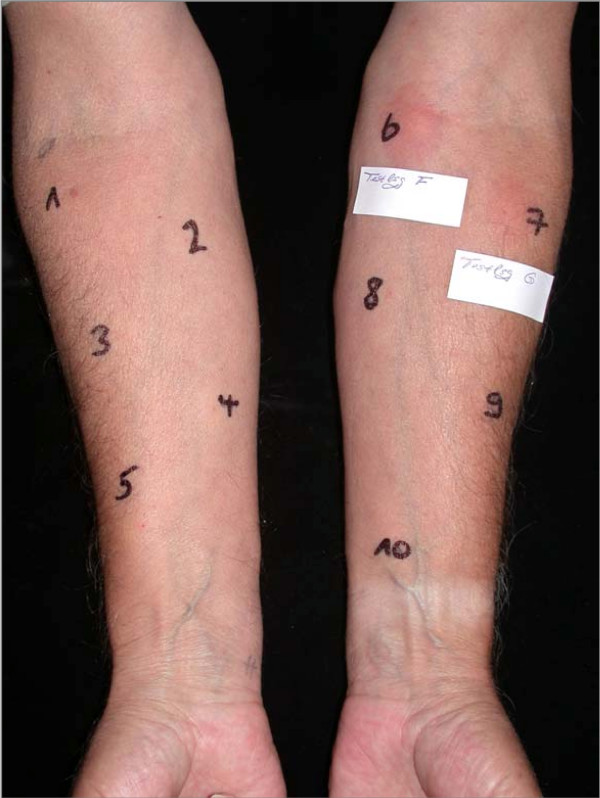
**Results of intradermal testing using the Sanofi Aventis Insuman^© ^test kit**. Protamine-containing test solutions (6 and 7) showed clear positive results 20 minutes after injections, while other components were negative.

Intradermal tests were performed with 0.05 ml of different standard insulins and with a *Lantus^© ^*test kit from Sanofi Aventis (Frankfurt/Main, Germany) on the volar forearm. Physiological saline and histamine (0.01% histamine solution; Bencard, Munich, Germany) served as controls. Table 1 shows the results of intradermal testing in detail. Figures 1 and 2 show positive intradermal testing with *Levemir^©^, Huminsulin basal^© ^Humalog^©^*, and *Lantus^© ^*(Fig. 1) and positive reactions against protamine-containing test solutions (Fig. 2).

**Table 1:** Substances used in intradermal testing

	Substance	20 minutes	24 hours	48 hours
1	***Levemir^© ^***(insulin glargine, m-cresol, glycerol)	+	++	++
2	***Huminsulin basal^© ^***(human insulin, m-cresol, phenol, glycerol, protamine)	+	++	++
3	***Lantus^© ^***(insulin glargine, m-cresol, glycerol)	+	++	++
4	***Actrapid penfill^© ^***(human insulin, m-cresol, glycerol)	+	++	++
5	***Insuman rapid^© ^***(human insulin, m-cresol)	+	++	++
6	***Berlinsulin H normal^© ^***(human insulin, phenol, protamine, glycerol)	+	++	++
7	***Insulin Novo semilente^© ^***(porcine insulin, methyl-4-hydroxybenzoate, natrium acetate)	+	++	++
8	***Humalog^© ^***(insulin lispro, m-cresol, glycerol, NaH_2_PO_4 _× H_2_O, zinc oxide)	+	+	+
9	***Novorapid^© ^***(insulin aspart, glycerol, m-cresol, phenol, NaH_2_PO_4 _× H_2_O)	+	+	+
10	***Apidra^© ^***(insulin glulisine, m-cresol, trometamol, polysorbate 20)	+	+	+
11	**Test solution A **(NaH_2_PO_4 _× H_2_O 2.1 mg, glycerol 85% 18.8 mg, phenol 0.6 mg, m-cresol 1.5 mg in aqua dest. ad 1.0 ml)	-	-	-
12	**Test solution B **(glycerol 85% 18.8 mg, phenol 0.6 mg, m-cresol 1.5 mg in aqua dest. ad 1.0 ml)	-	-	-
13	**Test solution C **(phenol 0.6 mg, m-cresol 1.5 mg in aqua dest. ad 1.0 ml)	+	-	-
14	**Test solution D **(phenol 0.6 mg in aqua dest. ad 1.0 ml)	+	-	-
15	**Test solution E **(m-cresol 1.5 mg in aqua dest. ad 1.0 ml)	+	-	-
16	**Test solution F **(protamine 0.1 mg, NaH_2_PO_4 _× H_2_O 2.1 mg, glycerol 85% 18.8 mg, phenol 0.6 mg, m-cresol 1.5 mg in aqua dest. ad 1.0 ml)	+	++	++
17	**Test solution G **(protamine 0.1 mg in aqua dest. ad 1.0 ml)	-	++	++
18	**Test solution H **(zinc chloride 0.06 mg, glycerol 85% 20 mg, m-cresol 2.7 mg in aqua dest. ad 1.0 ml)	-	-	-
19	**Test solution I **(glycerol 85% 20 mg, m-cresol 2.7 mg in aqua dest. ad 1.0 ml)	-	-	-
20	**Test solution J **(m-cresol 2.7 mg in aqua dest. ad 1.0 ml)	-	-	-
21	**Aqua dest.**	-	-	-
22	**NaCl 0.9%**	-	-	-
23	**Histamine 0.01%**	+	-	-

Test solutions A-J were obtained from the Sanofi Aventis Insuman^© ^test kit. Test results were noted 20 minutes, 24 hours, and 48 hours after injection. Interpretation of test results: -, no skin reaction; +, erythema and infiltrate with a diameter of <20 mm; ++, erythema and infiltrate with a diameter of >20 mm.

Patch testing of the same substances and of different local disinfectants was negative.

### Laboratory testing

Analysis of a blood sample showed normal islet cell antibodies (<1:10), elevated IgG antibodies against human insulin (56 U/ml; normal value, <1 U/ml), and elevated IgG antibodies against porcine insulin (12.4 ratio; normal value, <10.0). IgE antibodies against human and porcine insulin and against protamine were negative.

### Histology

**Figure 3:**
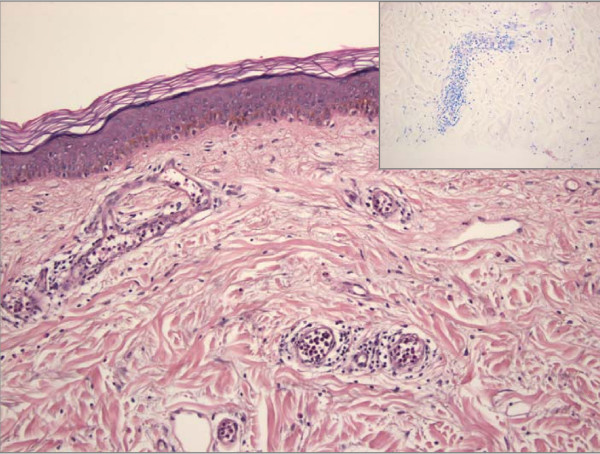
**Histologic slide of a skin biopsy obtained from an allergic skin reaction on the injection site: regular epidermis; congestion of different inflammatory cells in blood vessels with emission in the adjacent connective tissue of deeper dermal parts**. Hematoxylin/eosin staining, magnification ×200; inset: Giemsa staining, magnification ×200.

A skin biopsy taken from a plaque on an injection site of the abdominal wall showed an Arthus-type reaction (Fig. 3).

### Therapy

On day 1, we treated the patient with subcutaneous injections of human insulin (0.004, 0.01, 0.02, 0.04, 0.1, 0.2, 0.5, and 1.0 IU) using injection intervals of 30 minutes with a daily allowance of 1.874 IU. Fexofenadin (180 mg twice daily) was used as a concomitant medication as recommended by Grammer and coworkers [15]. On day 2, we injected 1.0, 2.0, 3.0, and 5 IU using injection intervals of 30 minutes. A daily allowance of 11 IU human insulin was reached. On day 3, we switched to the formerly incompatible insulin, *Lantus^©^*, given twice daily at a dose of 6 IU. Therapy was well-tolerated on all days with normoglycaemic values. On day 3, the local reactions decreased to slight cutaneous reactions of 2 mm in diameter. Up to the present time, the patient has tolerated this form of therapy and fexofenadin treatment was reduced to 180 mg daily, and then stopped completely, 6 months after desensitization.

## Discussion

Successful treatment of allergies due to insulin preparations has been reported during the last few years. In cases of hypersensitivity against protamine, the replacement of protamine-containing insulins by insulins without this additive is the simplest strategy to solve the problem. In patients in whom the insulin molecule itself causes local or systemic allergies, the management of these complications becomes much more difficult. Many authors have reported effective treatment using the insulin analogues, aspart and lispro, instead of human regular insulin [11,12,16]. Unfortunately, in our patient, intracutaneous testing of insulin lispro, insulin aspart, and insulin glulisine also caused an allergic test reaction. Therefore, a change to one of the less immunogenic insulins did not seem to be a promising option. Other groups have managed insulin allergies with continuous subcutaneous insulin infusions or with intravenously injected insulins [8,9,17]. In all cases, these forms of therapy were successful, but were in part associated with a restricted quality-of-life. In severe cases, a solitary pancreas transplantation was the last chance to treat a life-threatening insulin allergy [13,14].

According to cases reported by Wessbecher et al. [18] in 2001 and Barranco et al. [19] in 2003, we devised an ultra-rush treatment scheme using the subcutaneous administration of human insulin. After 3 days of therapy, our patient tolerated the formerly incompatible glargine insulin and showed only minimal local reactions at the injection site and which did not exceed a diameter of 2 mm.

The mechanism of tolerance induction in general and in our patient in particular still remains unclear. The most common type of insulin allergy is related to an IgE-mediated type I allergic reaction of the Coombs and Gell classification [2]. Less frequently, type III Arthus-type reactions have been reported [2]. In addition, insulin hypersensitivity can be related to a T-cell mediated type IV reaction. Our patient exhibited two different forms of hypersensitivity: 1) hypersensitivity against protamine and 2) hypersensitivity against the insulin molecule itself. As epicutaneous testing was completely negative, a T-cell mediated form of allergy seemed to be improbable. Histologic evaluation of a skin biopsy obtained from a local reaction proved an Arthus-type reaction, clearly indicating a type III reaction. Nevertheless, desensitization, such as performed in our patient and usually only successful in IgE-mediated type I reactions, was able to induce tolerance against formerly incompatible insulins.

## Conclusion

We would like to recommend insulin desensitization using an ultra-rush protocol with subcutaneous insulin applications as a rapid and easy method of treatment, even in cases in which intracutaneous testing is positive for several or all insulin preparations on-hand.

## Competing interests

The authors declare that they have no competing interests.

## Authors' contributions

CP, CSLM, DOH and WT were involved in drafting the manuscript. CP and DOH performed the allergological testing and desensitization while CSLM carried out the histologic analysis of the skin biopsy. All authors have read and approved the final manuscript.

## Consent

Written informed consent was obtained from the patient for publication of the case report and any accompanying images. A copy of the written informed consent is available for review by the Editor-in-Chief of this journal.
